# Comparative effectiveness of horticultural therapy modalities for cognitive function and depressive symptoms in older adults with cognitive impairment: Protocol for a systematic review and network meta-analysis

**DOI:** 10.1371/journal.pone.0351413

**Published:** 2026-06-11

**Authors:** Jingjing Yan, Weihua Li, Yingjie Shen, Qiyuan Lyu, Suwen Hu, Nan Xu

**Affiliations:** 1 School of Nursing, Chongqing Three Gorges Medical College, Chongqing, China; 2 Department of Nursing Science, Faculty of Medicine, University of Malaya, Kuala Lumpur, Malaysia; 3 School of Nursing, Jinan University, Guangzhou, China; 4 School of Medicine, Quzhou College of Technology, Zhejiang, China‌‌; Zhejiang Agriculture and Forestry University: Zhejiang A and F University, CHINA

## Abstract

**Introduction:**

Cognitive impairment in older adults is often accompanied by depressive symptoms, leading to reduced quality of life and increased risk of disability and mortality. Horticultural therapy has been increasingly used as a non-pharmacological intervention; however, its effectiveness may vary across different intervention modalities and delivery characteristics. Existing meta-analyses have primarily focused on overall effects and have not compared different horticultural therapy modalities. This study aims to evaluate and rank the comparative effectiveness of horticultural therapy delivered through different modalities using a network meta-analysis.

**Methods and analysis:**

Randomized controlled trials (RCTs) involving older adults with cognitive impairment will be included. The intervention group will receive horticultural therapy in addition to routine care, while the control group will receive routine care alone. Eight databases (PubMed, Embase, the Cochrane Central Register of Controlled Trials (CENTRAL), Web of Science, CINAHL, PsycINFO, China National Knowledge Infrastructure (CNKI), and Wanfang Database) will be searched from inception to May 2026. Interventions will be classified based on delivery characteristics, including environmental settings, cultivation substrates, and levels of participant engagement. Primary outcomes are cognitive function and depressive symptoms. Two reviewers will independently perform study selection, data extraction, and risk of bias assessment using the Cochrane Risk of Bias 2 tool. Pairwise meta-analysis and frequentist network meta-analysis will be conducted using the R (package “netmeta”), and treatment rankings will be estimated using surface under the cumulative ranking curve (SUCRA) values.

**Ethics and dissemination:**

Ethical approval is not required. Findings will be disseminated through a peer-reviewed publication.

**Trial registration:**

PROSPERO registration number: CRD420251185714.

## Introduction

With the accelerating aging of the global population, cognitive impairment has become an increasingly common condition among older adults [1]. It typically manifests as gradual declines in memory, attention, executive function, and emotional regulation, which may eventually compromise independent functioning and increase reliance on long-term care services [2]. As a result, cognitive impairment poses substantial challenges not only for affected individuals but also for their families and healthcare systems [3]. In clinical and community settings, cognitive impairment is frequently accompanied by depressive symptoms [4]. These emotional difficulties may be related to disease awareness, disruptions in social roles, and reduced opportunities for social participation [5,6]. The combination of cognitive impairment and depression has been linked to poorer functional results and decreased quality of life in later age [7]. Pharmacological treatments remain a common component of cognitive impairment management [8]. However, current research indicates that their benefits for cognitive and emotional outcomes are frequently limited, and questions about tolerance and long-term use remain [9,10]. In this setting, non-pharmacological therapies with the potential to improve cognitive function while also alleviating depression symptoms are receiving increased attention [11]. Therefore, exploring interventions that can improve cognitive function and relieve depressive mood at the same time has become the focus of research.

Horticultural therapy (HT) is a professionally guided intervention that seeks to improve physical and psychological well-being through plant-based and horticultural activities [12]. Although its clinical application dates back to the eighteenth century, HT was more formally developed into a systematic strategy during the twentieth century, particularly through the efforts of the American Horticultural Therapy Association [13]. From a mechanistic perspective, HT may promote cognitive function through structured, goal-directed activities that stimulate attention, memory, and executive functioning. Repetitive and task-oriented gardening activities may enhance procedural learning and cognitive engagement [14]. In addition, exposure to natural environments has been associated with stress reduction and emotional regulation, potentially alleviating depressive symptoms [15]. Functional engagement in meaningful activities may further support self-efficacy and behavioral activation in older adults with cognitive impairment [16,17]. Empirical studies suggest that HT is associated with improvements in mood, stress management, self-care abilities, and social interaction. Gardening activities, such as planting, pruning, and plant maintenance, may help establish daily routines, promote physical activity, and support functional independence [18]. Furthermore, HT provides multisensory stimulation and opportunities for social participation, which may enhance cognitive engagement and emotional stability in older adults with cognitive impairment [19,20]. Therefore, HT has gradually become an important part of non-pharmacological interventions for people with cognitive impairment, providing new evidence and practical directions for delaying functional degradation and improving quality of life.

Several recent systematic reviews and meta-analyses have evaluated the effectiveness of HT for depressive symptoms. For example, Kuo et al. conducted a meta-analysis examining the efficacy of HT for individuals with depressive disorders and reported improvements in depressive symptoms and functional outcomes [21]. Similarly, Chen et al. explored how intervention duration and frequency influence the effect of HT on depressive symptoms [22]. Although these studies provide important evidence regarding the therapeutic potential of horticultural interventions, they primarily focus on depressive symptoms in heterogeneous populations and evaluate HT as a single intervention category using traditional pairwise meta-analytic approaches. Horticultural therapy is a heterogeneous intervention comprising diverse modalities that differ in engagement mode, environmental setting, and cultivation substrate, which may influence therapeutic outcomes [13]. Furthermore, older adults with cognitive impairment represent a distinct clinical population with specific cognitive, psychological, and functional needs [23]. Evidence derived from general populations with depressive disorders may therefore not be directly applicable to this group. In addition, these studies did not use advanced methods, such as network meta-analysis, to evaluate comparative effectiveness across multiple modalities. Therefore, the current study does not replicate previous meta-analyses; instead, it addresses a distinct research question focused on comparative effectiveness across heterogeneous HT modalities in a specific clinical population.

Given the diversity of HT modalities and the limitations of existing pairwise meta-analyses, there remains a lack of comprehensive evidence comparing their relative effectiveness. A network meta-analysis enables the simultaneous comparison and ranking of multiple interventions by integrating both direct and indirect evidence, thereby overcoming the limitations of conventional pairwise meta-analyses. The proposed three-dimensional classification captures key therapeutic components that may influence cognitive and emotional outcomes, thereby allowing for more precise and clinically interpretable comparisons across heterogeneous interventions. To date, few network meta-analyses have systematically evaluated and compared the relative effectiveness of different HT modalities in improving both cognitive function and depressive symptoms among older adults with cognitive impairment. Therefore, this study aims to apply a network meta-analysis to compare and rank the effectiveness of different horticultural therapy modalities in improving cognitive and depression outcomes.

## Methods

This protocol is prepared in accordance with the Preferred Reporting Items for Systematic Review and Meta-Analysis Protocols (PRISMA-P) 2015 statement, and the completed review will be reported in accordance with the PRISMA 2020 guidelines. This protocol has been registered in the International Prospective Register of Systematic Reviews (PROSPERO; registration number: CRD420251185714). No amendments have been made since the initial registration.

### Study status

At the time of submission, the study has not yet generated results. Literature screening and data extraction have not been completed. Data collection is expected to be completed by June 2026, and the final results are anticipated by December 2026.

### Eligibility criteria

#### Type of participants.

We will include adults aged 60 years or older with a clinical diagnosis of cognitive impairment, including mild cognitive impairment and mild-to-moderate dementia, as defined by recognized diagnostic criteria. Differences in diagnostic criteria and severity levels (e.g., mild cognitive impairment vs. mild or moderate dementia) will be documented and treated as potential effect modifiers in subgroup or meta-regression analyses where data permit. Participants should be clinically stable and engage in horticultural activities, as determined by the original study authors. No restrictions will be placed on the duration of cognitive impairment. Individuals with severe physical illnesses (e.g., malignancies, organ failure) or pronounced psychiatric and behavioral disturbances will be excluded. In addition, those with significant sensory deficits, such as blindness or severe hearing loss, that would preclude participation in horticultural activities will also be excluded.

#### Type of intervention.

This review will include interventions that integrate HT with usual care for older adults with cognitive impairment. To ensure conceptual clarity and enable valid network comparisons, HT interventions will be categorized according to their media and implementation characteristics. Three predefined classification dimensions will be applied to categorize HT interventions.

First, the mode of engagement will be classified as active horticultural activities or passive horticultural exposure. Active engagement will be defined as activities that primarily involve direct physical interaction with plants (e.g., planting, watering, pruning). In contrast, passive engagement will refer to activities that primarily involve observation or sensory exposure (e.g., viewing plants, smelling flowers). Interventions will be classified as “primarily active” if more than 50% of the session time is spent in active participation. Second, the activity settings will be divided into indoor and outdoor horticulture. Indoor settings will include enclosed or semi-enclosed environments (e.g., rooms, greenhouses), while outdoor settings will refer to open natural environments such as gardens or parks. Greenhouse-based interventions will be classified as indoor unless clearly described as open-air or outdoor-integrated environments. Finally, cultivation techniques will be categorized as soil-based or soilless, including hydroponic and substrate-based systems. For interventions involving mixed cultivation media (e.g., both soil-based and soilless systems), classification will be based on the predominant cultivation method used during the intervention.

**Fig 1:**
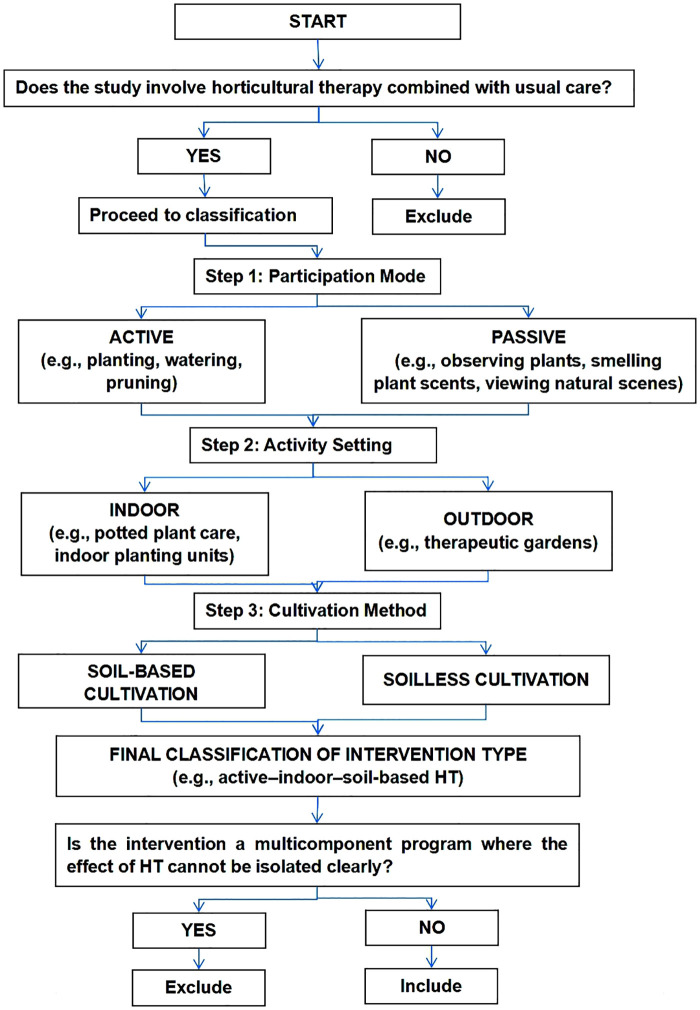
Flowchart of the classification of horticultural therapy interventions.

If no dominant component can be identified, the study will be categorized by the authors’ primary therapeutic focus. Intervention categories will be defined a priori based on conceptual and clinical relevance, and detailed classification criteria will be developed to ensure consistency and reproducibility. Sensitivity analyses will be conducted to assess the robustness of the classification strategy. Fig 1 shows the classification of HT interventions.

The intervention components (e.g., activity mode, cultivation medium, and setting) were not factorially designed or independently varied across trials. As a result, the assumptions required for component network meta-analysis (CNMA), particularly the additivity assumption, are unlikely to be satisfied. Therefore, a conventional network meta-analysis was considered more appropriate for comparing the relative effectiveness of different HT modalities. This network meta-analysis will use a standard intervention-level approach, with each intervention node representing a predefined set of HT characteristics. To reduce overlap between intervention categories, each study will be assigned to a single treatment node based on the overall characteristics of the HT intervention. This single-node assignment is required to ensure the validity of the network meta-analysis framework. However, given that many horticultural interventions include mixed or overlapping characteristics, explicit operational decision rules are necessary to ensure consistent and reproducible classification across studies.

To enhance reproducibility, explicit decision rules will be applied when assigning studies to intervention nodes. When interventions have mixed characteristics, classification will follow a hierarchical approach based on the primary therapeutic intent and the dominant intervention features reported by the study authors. Specifically, priority will be given in the following order: (1) mode of engagement (active vs passive), as this reflects the level of cognitive and physical involvement; (2) intervention setting (indoor vs outdoor), reflecting environmental exposure; and (3) cultivation substrate (soil-based vs soilless), reflecting technical implementation. When multiple components are present, the component with the greatest intensity (e.g., duration, frequency, or proportion of session time) will be considered dominant. If insufficient detail is provided, consensus will be reached through discussion between reviewers. Representative examples of classification decisions will be provided in S1 Table.

#### Comparison.

Variations in usual care will be explored through sensitivity analyses. The comparator conditions will include either usual care, such as pharmacological management, cognitive training, health education, routine nursing care, or prevention of complications; or an alternative form of HT using a different medium, as defined in the intervention classification framework of this review. Usual care may vary across studies and will be carefully documented to evaluate its potential impact on treatment comparisons. When HT with a different medium serves as the comparator, clear documentation of the medium category and intervention characteristics will be required. Studies employing multi-component interventions in which the independent effect of HT cannot be isolated will be excluded.

#### Type of outcomes.

It is expected that the findings will primarily concentrate on cognitive function and depression. The focus of the outcome will be cognitive function, which can be evaluated with a recognized cognitive assessment tool, such as the Mini-Mental State Examination, Montreal Cognitive Assessment, the Subjective Cognitive Decline Questionnaire, Memory Impairment Screen, Mini-Addenbrooke’s Cognitive Examination, Addenbrooke’s Cognitive Examination-III, Clock-Drawing Test, Quick Screen for Mild Cognitive Impairment, or Repeatable Battery for the Assessment of Neuropsychological Status [24]. Depression will be assessed using validated instruments, including the Geriatric Depression Scale (GDS), the Cornell Scale for Depression in Dementia (CSDD), and the Depression–Anxiety–Stress Scale-21 (DASS-21) [25–27]. Depending on how the instruments are scored, higher scores usually indicate more severe depressive symptoms (S2 Table). All outcome measures will be standardized using SMDs, and score directions will be aligned so that higher values consistently reflect either improvement or deterioration, depending on the outcome. Where available, secondary outcomes such as quality of life and functional status will also be extracted and narratively summarized to provide a more comprehensive understanding of intervention effects.

#### Type of studies.

We will focus on randomized controlled trials, quasi-randomized trials, and controlled clinical trials that investigate how horticulture treatment affects cognitive function or depressive symptoms in older persons with cognitive impairment. Eligible studies must provide a comprehensive description of both the intervention and comparator conditions, as well as sufficient quantitative outcome data to be included in the intended network meta-analysis. Due to potential methodological differences across study designs, randomized controlled trials will constitute the principal evidence base for the primary network meta-analysis. The inclusion of controlled clinical trials and quasi-randomized trials will improve the comprehensiveness of evidence collection, and sensitivity analyses will be used to evaluate their impact on pooled estimates. Sensitivity analyses will be conducted by excluding non-randomized or quasi-randomized studies to assess the robustness of the findings. Where sufficient data are available, subgroup analyses stratified by study design will also be considered. Observational studies, single-group pre–post designs, case reports, qualitative studies, reviews, mixed-methods studies without separable quantitative outcomes, animal studies, and trials involving complex multicomponent interventions in which the independent effects of HT cannot be determined will be excluded. Studies without data on cognitive or depressive outcomes will also be excluded.

### Data sources and search strategy

A comprehensive systematic literature search will be conducted between 1 December 2025 and 31 May 2026. The following electronic databases will be searched from inception to May 2026: PubMed, Embase, Cochrane Central Register of Controlled Trials (CENTRAL), Web of Science, CINAHL, PsycINFO, and major Chinese databases, including China National Knowledge Infrastructure (CNKI) and WanFang. In addition, clinical trial registries such as ClinicalTrials.gov will be searched to identify ongoing or unpublished studies. No restrictions will be applied on publication year, but only studies published in English or Chinese will be included. Language restrictions may introduce language bias; however, this approach was adopted to ensure feasibility and accurate interpretation of the included studies. The search strategy will combine controlled vocabulary terms (e.g., MeSH) and free-text keywords related to: (1) cognitive impairment (e.g., “cognitive dysfunction,” “mild cognitive impairment,” “dementia”); (2) horticultural therapy (e.g., “horticulture,” “gardening,” “therapeutic horticulture”), and (3) older adults (e.g., “aged,” “elderly”). The PubMed search strategy is outlined in Online Supplements (S1 Appendix), with the corresponding search approach for the Chinese databases. The full search strategies for each database will be adapted as appropriate. Additionally, reference lists of relevant systematic reviews and included studies will be manually searched to identify any additional eligible trials.

### Study selection

**Fig 2:**
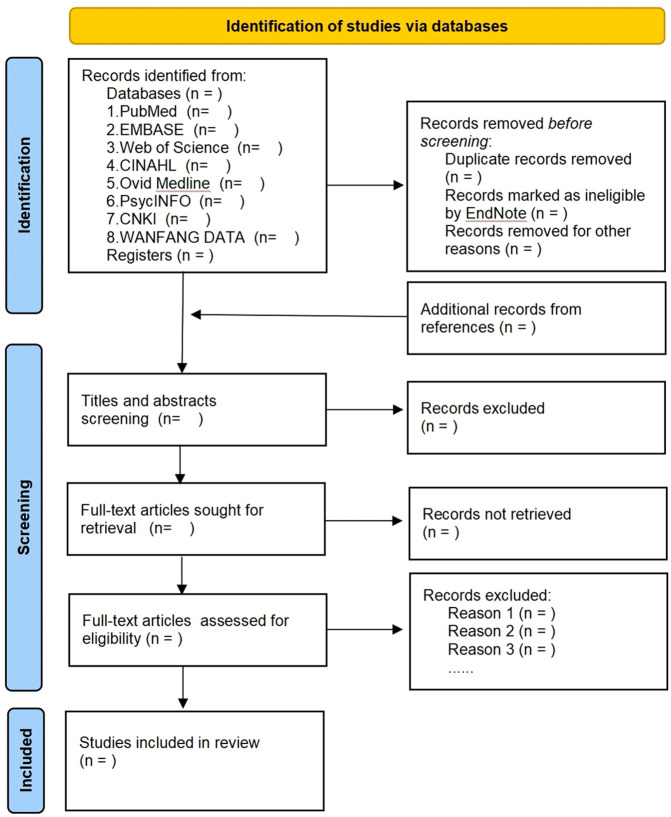
Flowchart of the literature selection process.

To facilitate management, all retrieved records will be imported into EndNote, and duplicate entries will be eliminated. Studies published in English and Chinese will be included. Reviewers proficient in both languages will independently conduct study selection and data extraction to ensure accurate interpretation of non-English studies. Two reviewers will independently review the titles and abstracts of the remaining studies to remove those that evidently fail to satisfy the inclusion criteria. Then, the results of the first screening will be evaluated again. The same two reviewers will independently assess the full texts to establish final eligibility. Disagreements will be addressed by consulting a third reviewer, followed by a discussion to achieve consensus. Fig 2 illustrates the study selection process. To ensure methodological consistency across English and Chinese studies, identical eligibility criteria, data extraction procedures, and risk-of-bias assessment standards will be applied regardless of publication language. Duplicate publications across Chinese and English databases will be carefully screened and removed.

### Data extraction

First, all research team members will develop a standardized data extraction template through group discussion. Subsequently, two reviewers will independently and thoroughly examine the full texts of eligible studies and extract relevant information using the predefined template. The authors and year of publication, sample size and participant characteristics (such as age and gender), the degree of cognitive impairment, the media used in HT, the intervention frequency and duration, outcome indicators, and measurement instruments are among the variables that may be extracted from the data. When multiple time points are reported, post-intervention outcome data will be extracted preferentially. If post-intervention data are unavailable, data from the longest follow-up will be used. Results that are categorical or continuous will be taken directly and placed in a specific Excel file (S1 File). One reviewer will check the extracted data against the other's, and any differences will be resolved through discussion.

### Risk-of-bias (RoB) assessment

The risk of bias of randomized controlled trials will be assessed using the revised Cochrane Risk of Bias tool (RoB 2) [28]. This instrument assesses five primary areas of potential bias: randomization bias, intervention deviation bias, missing outcome data bias, measurement bias, and reported result selection bias. A “low risk,” “some concerns,” or “high risk” rating will be assigned to each domain. The overall RoB rating for each study will follow the same set of categories. For quasi-randomized and controlled clinical trials, the Risk of Bias in Non-Randomized Studies of Interventions (ROBINS-I) tool will be employed. Two reviewers will independently perform the assessments and subsequently compare their judgments. A third reviewer will be consulted to resolve any disagreements. To further quantify the agreement amongst raters, we shall compute Cohen's kappa coefficient. Particular attention will be paid to methodological reporting quality in non-English studies, including randomization procedures, allocation concealment, and completeness of outcome reporting. When reporting is unclear, study authors will be contacted where feasible, and unclear domains will be conservatively judged according to RoB guidance.

### Statistical analysis

This study will follow methodological guidance for conducting network meta-analyses in systematic reviews to ensure transparency and reproducibility [29]. Given the emerging nature of horticultural therapy research, the feasibility of conducting a network meta-analysis will depend on the final number, connectivity, and comparability of eligible studies. If the available evidence is insufficient to support a robust network structure, findings will be synthesized narratively or using pairwise meta-analysis where appropriate.

#### Pairwise and network meta-analysis.

This review adopts a traditional network meta-analysis framework, in which each unique combination of intervention characteristics is treated as a distinct intervention node, rather than estimating the independent effects of individual components. A network meta-analysis will be conducted only if the evidence network is connected through at least one common comparator (e.g., usual care, waitlist control, or non-horticultural activity). Before conducting the network meta-analysis, the geometry and connectivity of the evidence network will be carefully assessed. A network meta-analysis will be performed only if sufficient direct or indirect comparisons are available among intervention nodes. Intervention categories with insufficient studies or disconnected structures will be merged where conceptually appropriate, or narratively summarized rather than quantitatively synthesized. The validity of indirect comparisons relies on the transitivity assumption. The transitivity assumption will be tested by examining the distribution of potential effect modifiers across treatment comparisons, such as baseline age, severity of cognitive impairment, intervention duration, and outcome-measurement instruments. Only trials deemed sufficiently comparable with respect to these characteristics will be included in the network meta-analysis.

Pairwise meta-analyses will be conducted using Review Manager version 5.3. Standardized mean differences and the corresponding 95% confidence intervals will be calculated for continuous outcomes. Heterogeneity will be assessed using both the χ² test and the I² statistic. I² values of 25%, 50%, and 75% will be considered low, moderate, and high heterogeneity, respectively [30]. Substantial heterogeneity (I² > 50%) will be explored through subgroup analyses and sensitivity analyses. A frequentist random-effects network meta-analysis will be conducted using the R (package “netmeta”). The random-effects model is chosen to account for anticipated clinical and methodological heterogeneity across studies. Between-study heterogeneity will be quantified, and model robustness will be assessed through sensitivity analyses [31]. The surface under the cumulative ranking curve (SUCRA) values with their 95% confidence intervals will be calculated to rank the relative effectiveness of HT interventions for each primary outcome, including cognitive and emotional outcomes. Ranking heat plots will be generated using appropriate visualization functions in R. SUCRA rankings will be interpreted cautiously in conjunction with effect estimates and the assessed quality of evidence [32,33].

#### Dealing with missing data.

We will contact the corresponding authors to obtain the necessary details for missing data, including outcome scores and information on the cultivation media. When possible, we will use available information to estimate the missing data if no response is received. The possible impact of missing data on the reliability of the findings will be assessed using a sensitivity analysis.

#### Assessment of publication bias.

Publication bias and small-study effects will be assessed using comparison-adjusted funnel plots in the context of network meta-analysis. If at least 10 studies are included in the network meta-analysis, publication bias will be assessed using comparison-adjusted funnel plots, complemented by Egger’s regression test. Stata (version 15.0) will be used for the statistical analyses [34].

#### Assessment of inconsistency and subgroup analysis.

Local inconsistency within each closed loop will be assessed using the loop-specific approach, and node-splitting analyses will be performed to compare direct and indirect evidence for key intervention comparisons. Global inconsistency across the entire network will be evaluated using the design-by-treatment interaction model. All analyses will be conducted in Stata (version 15.0) [35,36]. If significant inconsistency is detected, potential sources will be explored through sensitivity analyses and meta-regression, and results will be interpreted with caution. Subgroup analyses will be performed using the same analytic procedures. Prespecified subgroup analyses will be conducted based on clinically relevant and methodologically important variables. These subgroup classifications will include: (1) severity of cognitive impairment (mild cognitive impairment vs mild-to-moderate dementia); (2) intervention duration (≤8 weeks vs > 8 weeks); (3) intervention frequency (≤1 session/week vs > 1 session/week); (4) participant age (<75 years vs ≥ 75 years); (5) mode of horticultural engagement (active vs passive); and (6) study design (randomized vs non-randomized studies). Subgroup thresholds were determined a priori based on clinical relevance and distributions commonly reported in previous horticultural therapy studies. Subgroup analyses will be conducted using separate network meta-analyses or network meta-regression where sufficient studies are available. A minimum of three studies per subgroup category will generally be required to support quantitative subgroup comparisons.

#### Sensitivity analysis.

Sensitivity analyses will be conducted for all outcome measures to assess the robustness of the findings. These analyses will include, but will not be limited to, the exclusion of studies with a high risk of bias and those with incomplete outcome data. Furthermore, to account for potential clinical heterogeneity and variability in comparator conditions, sensitivity analyses will be done by excluding trials in which the control group received additional structured therapeutic activities, such as formal cognitive training or exercise programs, in addition to usual care. The influence of these exclusions on effect estimates, network consistency, and overall model fit will be analyzed. Consistency of findings across sensitivity analyses will be used to evaluate the stability of the results and the plausibility of the underlying assumptions of the network meta-analysis. Additional sensitivity analyses will be conducted by excluding studies published in Chinese-language journals to assess the influence of this exclusion on pooled effect estimates, network consistency, and overall conclusions.

#### Quality of evidence.

The quality of evidence for all outcomes will be assessed using the Grading of Recommendations Assessment, Development and Evaluation (GRADE) framework. The evaluation will focus on the research’s limitations, imprecision, heterogeneity, inconsistency, indirectness, and publication bias [37]. Where applicable, the Confidence in Network Meta-Analysis (CINeMA) framework will be used to assess the certainty of the evidence across the network.

## Discussion

The growing issue of cognitive impairment in aging populations highlights an urgent need for effective and scalable non-pharmacological therapies. Although previous meta-analyses have examined HT for depressive symptoms, these studies primarily evaluated HT as a single intervention and did not compare different HT modalities [21,22]. HT combines physical activity, multisensory engagement, and interaction with natural environments, which are hypothesized to impact cognitive and emotional outcomes in older individuals [19]. However, it is unclear how these potential advantages vary across HT approaches. HT encompasses a wide range of aspects, including participant participation levels, environmental settings, and cultivation methods [13]. Previous studies have used diverse methodologies and outcome measures, resulting in fragmented and sometimes inconsistent findings. The results of this study are expected to provide a comparative synthesis of available data regarding multiple HT methods, potentially informing future intervention design and research objectives.

Several additional limitations should be considered when interpreting the findings of this study. First, the robustness of the network meta-analysis depends on the connectivity of the evidence network. If few head-to-head trials are available, the network may rely heavily on indirect comparisons, reducing the precision of effect estimates. Second, clinical heterogeneity may arise from variations in usual care across studies, including differences in pharmacological treatments, cognitive training, and routine nursing care, which may influence relative treatment effects. Third, although randomized controlled trials will form the primary evidence base, the inclusion of quasi-randomized and controlled clinical trials may introduce additional risk of bias. Sensitivity and subgroup analyses will be conducted to assess the impact of study design on the results.

An additional limitation is the restriction to studies published in English and Chinese, which may introduce language bias. Studies published in other languages may have been missed, potentially leading to incomplete evidence coverage. Because studies with positive findings are more likely to be published in widely accessible languages, this may result in an overestimation of treatment effects. Furthermore, excluding studies from other linguistic and regional contexts may limit the generalizability of the findings and reduce the diversity of intervention settings represented in the network.

Despite these limitations, this study has several strengths. It will be among the early network meta-analyses to comprehensively compare different HT modalities in older adults with cognitive impairment. The use of predefined intervention classification criteria and planned sensitivity analyses is expected to enhance the transparency and robustness of the findings. In addition, the network meta-analysis approach enables the simultaneous comparison and ranking of multiple interventions, providing more detailed insights into the relative effectiveness of different HT characteristics.

From a clinical perspective, HT modalities may differ in their suitability for specific patient groups and care settings. Active, outdoor, soil-based interventions may offer greater cognitive stimulation and physical engagement, making them more suitable for individuals with mild cognitive impairment or preserved functional capacity. In contrast, passive or indoor-based interventions may be more appropriate for individuals with moderate impairment or limited mobility. In long-term care settings, simplified indoor horticultural activities may be more feasible to implement. Tailoring HT modalities to patient characteristics and care contexts may therefore enhance both feasibility and therapeutic effectiveness.

Future research should focus on developing standardized HT protocols to improve consistency across studies. High-quality head-to-head randomized controlled trials are needed to compare different HT modalities directly. In addition, longer follow-up periods should be incorporated to assess the sustainability of intervention effects. These efforts will help strengthen the evidence base and support the integration of HT into the management of cognitive impairment.

## Supporting information

S1 Checklist PRISMA-P (Preferred Reporting Items for Systematic review and Meta-Analysis Protocols) 2015 checklist: recommended items to address in a systematic review protocol*. (DOCX)

S1 Table Operational classification of horticultural therapy interventions across engagement mode, setting, and cultivation medium, with illustrative coding examples. (DOCX)

S2 Table Outcome measurement instruments. (DOCX)

S1 Appendix A draft search strategy. (DOCX)

S1 File Data extraction form. (DOCX)
