# Supplementary material for: Comparative effectiveness of horticultural therapy modalities for cognitive function and depressive symptoms in older adults with cognitive impairment: Protocol for a systematic review and network meta-analysis
Source: PLoS One. 2026 Jun 11;21(6):e0351413. doi: 10.1371/journal.pone.0351413 (PMC13257980; doi:10.1371/journal.pone.0351413)
Supplement: S1 Table — (DOCX) [file pone.0351413.s002.docx]

**S1 Table. Operational classification of horticultural therapy interventions across engagement mode, setting, and cultivation medium, with illustrative coding examples.**

| **Study** | **Node** | **Engagement** | **Setting** | **Medium** | **Coding Basis** |
| --- | --- | --- | --- | --- | --- |
| Chen et al., 2020 | Passive–Indoor–Soil | \| Passive  (participants observed plants and engaged in sensory exposure only) \| \| --- \| | \| Indoor  (activity room with potted plants) \| \| --- \| | \| Soil-based \| \| --- \| | >80% sessions involved plant observation in indoor room with potted soil plants |
| Wang et al., 2019 | Active–Indoor–Soilless | Active  (hands-on hydroponic planting activities) | Indoor  (greenhouse setting) | Soilless (hydroponics) | Main intervention component was hydroponic planting conducted indoors |
| Zhang et al., 2022 | Passive–Outdoor–Soil | Passive  (guided garden walks and plant observation) | Outdoor  (park environment) | Soil-based | Intervention focused on guided outdoor garden exposure rather than cultivation |
| ...... |  |  |  |  |  |

Note: Classification was based on the dominant intervention characteristics reported in each study. When mixed components were present, priority was assigned in the following order: engagement mode (active vs passive), setting (indoor vs outdoor), and cultivation medium (soil vs soilless). The dominant component was determined based on the proportion of session time, intervention intensity, and primary therapeutic intent as described by the study authors.
