# Supplementary material for: Comparative effectiveness of horticultural therapy modalities for cognitive function and depressive symptoms in older adults with cognitive impairment: Protocol for a systematic review and network meta-analysis
Source: PLoS One. 2026 Jun 11;21(6):e0351413. doi: 10.1371/journal.pone.0351413 (PMC13257980; doi:10.1371/journal.pone.0351413)
Supplement: S2 Table — (DOCX) [file pone.0351413.s003.docx]

S3 Table. Outcome measurement instruments. (docx)

| **Study** | **Outcome** | **Instrument** | **Score range** |
| --- | --- | --- | --- |
| 1 |  |  |  |
| 2 |  |  |  |
| 3 |  |  |  |
| 4 |  |  |  |
| 5 |  |  |  |
| 6 |  |  |  |
| ...... |  |  |  |
