# Supplementary material for: Comparative effectiveness of horticultural therapy modalities for cognitive function and depressive symptoms in older adults with cognitive impairment: Protocol for a systematic review and network meta-analysis
Source: PLoS One. 2026 Jun 11;21(6):e0351413. doi: 10.1371/journal.pone.0351413 (PMC13257980; doi:10.1371/journal.pone.0351413)
Supplement: S1 Appendix — (DOCX) [file pone.0351413.s004.docx]

**S1 Appendix. Search strategy for PubMed**

| 1 | #1 | ("Cognitive Impairment"[Mesh] OR "Dementia"[Mesh] OR "Alzheimer Disease"[Mesh] OR cognit* impair*[tiab] OR cognit* declin*[tiab] OR dement*[tiab] OR alzheimer*[tiab]) |
| --- | --- | --- |
| 2 | #2 | ("Horticultural Therapy"[Mesh] OR horticultural therap*[tiab] OR gardening therap*[tiab] OR therapeutic horticultur*[tiab]) |
| 3 | #3 | ("Aged"[Mesh] OR older adult*[tiab] OR elderly[tiab] OR older people[tiab]) |
| 4 | #4 | #1 AND #2 AND #3 |

Note: “*” to include all derivatives of that word or concept.

**S1 Appendix. Search strategy for Chinese databases**

| 1 | #1 | (SU = 痴呆 OR SU = 阿尔茨海默病 OR SU = 认知障碍 OR SU = 认知功能障碍 OR SU = 轻度认知障碍) |
| --- | --- | --- |
| 2 | #2 | (SU = 园艺治疗 OR SU = 园艺疗法 OR SU = 园艺活动 OR SU = 治疗性园艺) |
| 3 | #3 | (SU = 老年人 OR SU = 老年 OR SU = 高龄者) |
| 4 | #4 | #1 AND #2 AND #3 |

Note:

1. SU = title/abstract/keywords
