# Supplementary material for: Comparative effectiveness of horticultural therapy modalities for cognitive function and depressive symptoms in older adults with cognitive impairment: Protocol for a systematic review and network meta-analysis
Source: PLoS One. 2026 Jun 11;21(6):e0351413. doi: 10.1371/journal.pone.0351413 (PMC13257980; doi:10.1371/journal.pone.0351413)
Supplement: S1 File — (DOCX) [file pone.0351413.s005.docx]

| **Section** | **Variable** | **Description / Coding** | **Extracted Data** | | |
| --- | --- | --- | --- | --- | --- |
|  |  |  | **study1** | **study2** | **......** |
| Study Identification | First author | First author of the study |  |  |  |
|  | Year | Publication year |  |  |  |
|  | Country | Country where study conducted |  |  |  |
|  | Journal | Journal name |  |  |  |
| Study Characteristics | Study design | RCT / quasi-RCT / controlled clinical trial |  |  |  |
|  | Study setting | Community / hospital / nursing home |  |  |  |
|  | Sample size (total) | Total number of participants |  |  |  |
|  | Sample size (intervention) | Number in intervention group |  |  |  |
|  | Sample size (control) | Number in control group |  |  |  |
| Participant Characteristics | Mean age | Mean age of participants |  |  |  |
|  | Age range | Age range if reported |  |  |  |
|  | Gender | % female / male |  |  |  |
|  | Cognitive impairment type | MCI / dementia / other |  |  |  |
|  | Severity of cognitive impairment | Mild / moderate / severe |  |  |  |
| Intervention Characteristics | Intervention name | Description of horticultural therapy |  |  |  |
|  | Engagement mode | Active / passive horticulture |  |  |  |
|  | Setting | Indoor / outdoor |  |  |  |
|  | Cultivation medium | Soil / hydroponic / other substrate |  |  |  |
|  | Intervention frequency | Sessions per week |  |  |  |
|  | Session duration | Minutes per session |  |  |  |
|  | Total intervention duration | Weeks of intervention |  |  |  |
|  | Facilitator | Therapist / nurse / trained staff |  |  |  |
| Comparator | Comparator type | Usual care / alternative HT / other |  |  |  |
|  | Description of control | Description of control intervention |  |  |  |
| Outcomes | Cognitive function outcome | Name of scale (e.g., MMSE, MoCA) |  |  |  |
|  | Depression outcome | Scale used (e.g., GDS, CSDD, DASS-21) |  |  |  |
| Outcome Data | Cognitive score (baseline) | Mean ± SD |  |  |  |
|  | Cognitive score (post-intervention) | Mean ± SD |  |  |  |
|  | Depression score (baseline) | Mean ± SD |  |  |  |
|  | Depression score (post-intervention) | Mean ± SD |  |  |  |
| Follow-up | Follow-up time | Weeks or months |  |  |  |
| Risk of Bias | Randomization | Low / some concerns / high |  |  |  |
|  | Allocation concealment | Yes / No / unclear |  |  |  |
|  | Blinding | Yes / No / unclear |  |  |  |
|  | Missing outcome data | Yes / No / unclear |  |  |  |
| Additional Notes | Funding | Funding source |  |  |  |
|  | Conflict of interest | Reported COI |  |  |  |
|  | Notes | Other relevant information |  |  |  |
| Network Meta-analysis | Intervention node | Network node classification |  |  |  |
|  | Comparator node | Comparator category |  |  |  |
|  | Outcome time point | Immediate post-intervention / follow-up |  |  |  |
